# Artificial Intelligence vs Visual Assessment of Calcified Plaque in Coronary Artery Using Optical Coherence Tomography

**DOI:** 10.1016/j.jacadv.2022.100080

**Published:** 2022-08-31

**Authors:** Yuki Katagiri, Yuichiro Hosoi, Hiroki Bota, Ken Kuroda, Yutaro Kasai, Kohei Ishikawa, Naoyuki Semba, Kazumasa Yamasaki, Tomoyuki Tani, Seiji Yamazaki

Severe calcification limits stent expansion, and percutaneous coronary intervention (PCI) in calcified lesions is associated with a higher rate of revascularization and myocardial infarction. To facilitate stent expansion, intravascular imaging is used to evaluate the necessity for prior lesion modification. In optical coherence tomography (OCT), a calcium scoring system was developed to predict stent underexpansion, indicating which lesions would benefit from plaque modification before stent implantation.^1^ Recently, plaque characterization via OCT using artificial intelligence (AI) has been developed, which would improve objectivity and reproducibility compared with visual assessment.^2^ However, the accuracy of quantitative assessment of calcified plaque by AI compared with visual assessment is yet to be evaluated in the real-world clinical setting.

In this retrospective, single-center study, we compared AI and visual assessments of calcified plaque in preprocedural OCT images. The study protocol was approved by the Institutional Review Board of our hospital. All patients undergoing OCT-guided PCI between July 2015 and March 2022 were screened. Lesions with in-stent restenosis, OCT uncrossability, or suboptimal OCT imaging (eg, incomplete flush) were excluded from the study. Preprocedure, the automatic OCT pullback was performed using Dragonfly OPTIS or Dragonfly OpStar (both Abbott) with contrast injection at a pullback speed of 36 mm/s and frame rate of 180 frames/s. AI measurement of calcification angle, thickness, and length was performed using Ultreon software (version 1.0, Abbott). The Ultreon is the first commercially available AI that enables automatic online quantification of calcification in OCT images. It is based on deep learning that uses convolutional neural networks, trained by more than 7,000 OCT images with expert annotation as ground truth in its development. Visual assessment was performed quantitatively using QIvus (Research Edition 3.1.18.0, Medis Medical Imaging Systems BV) according to the standard guidelines^3^ by 2 independent interventional cardiologists and reviewed by a third reader who were all kept blinded to the AI measurement. Any disagreement between the observers was resolved through consensus. The statistical analysis was performed on a per lesion basis. In case of multiple calcium deposits, the calcium deposit with the largest maximal calcium angle was chosen to be representative of each lesion.

In total, 227 lesions in 192 patients were included in the study. The mean age was 72.1 ± 12.0 years, and 135 (70.3%) patients were male. Diabetes was present in 73 patients (38.0%). There were 63 (32.8%) patients with an estimated glomerular filtration rate <60 mL/min/1.73 m^2^ and 36 (18.8%) patients treated with hemodialysis.

**Figure 1:**
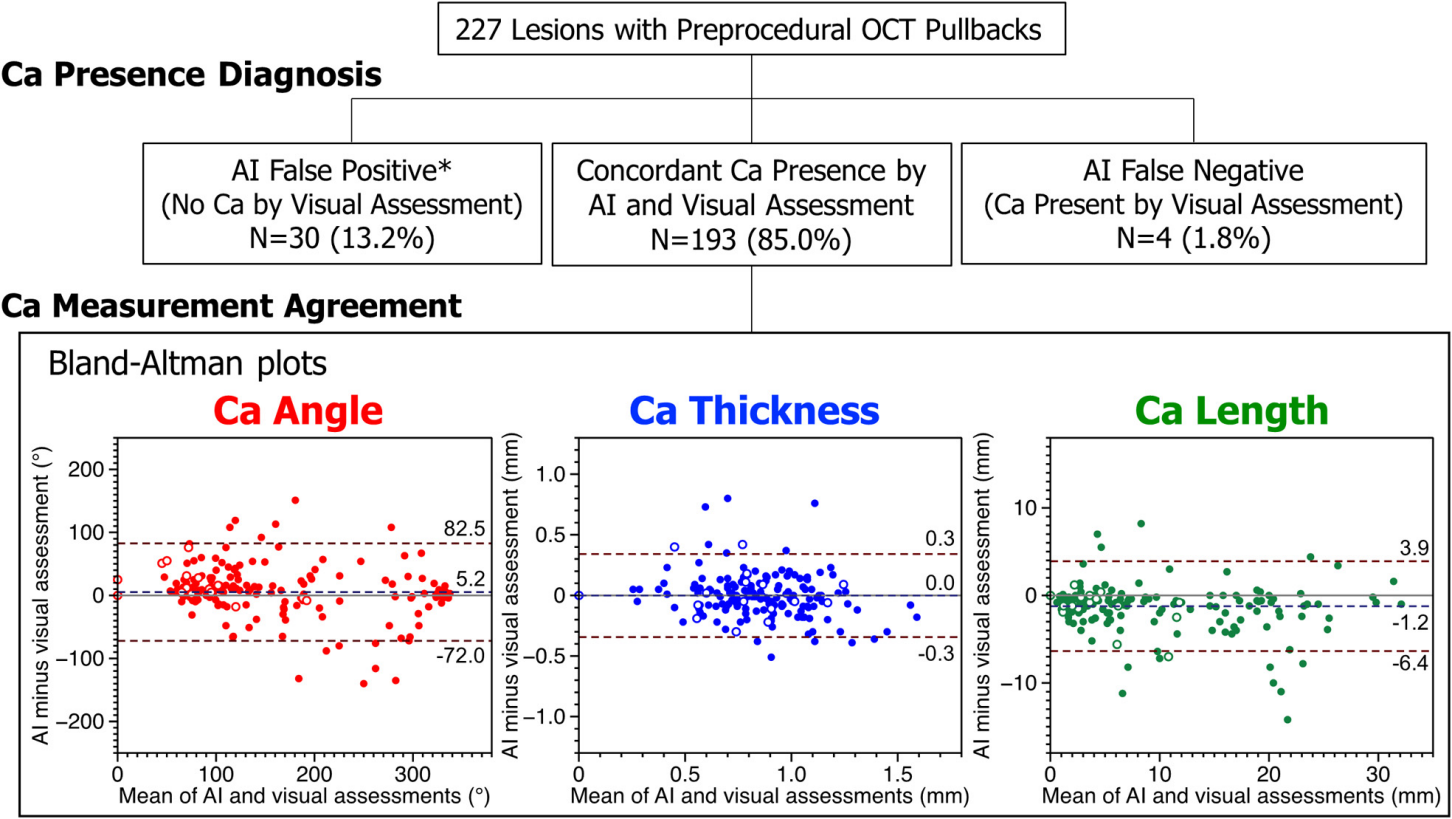
**Comparison Between AI and Visual Assessments of Calcified Plaque Using OCT** **(Upper row)** The agreement between AI and visual assessments in the diagnosis of Ca presence is observed in 85.0% of the lesions studied. ∗ACS as an indication for PCI is an independent predictor of AI-false positive. **(Lower row)** Bland-Altman plots comparing AI and visual assessments of the quantitative Ca measurements in lesions with concordant Ca presence diagnosis. Mean bias and 95% limits of agreement are shown by the dashed lines. White circles indicate ACS lesions. ACS = acute coronary syndrome; AI = artificial intelligence; Ca = calcium; OCT = optical coherence tomography; PCI = percutaneous coronary intervention.

In 30 (13.2%) lesions, only AI detected calcification (AI false positive), whereas lesions with AI-false negative (ie, calcification recognized only by visual assessment) were minor (4 [1.8%] lesions) (Figure 1). The multivariate logistic regression analysis adjusted for clinically relevant variables indicated that acute coronary syndrome as an indication for PCI was independently associated with AI-false positives (adjusted odds ratio: 6.47 [95% CI: 2.42-17.28]; *P* < 0.001). In lesions where calcification presence was in agreement between AI and visual assessments, there was a significant positive correlation between AI and visual assessments regarding calcification angle, thickness, and length (Pearson’s r = 0.930, 0.892, and 0.951, respectively [all *P* < 0.001]). The Bland-Altman plots indicated that bias was minimal in all these 3 parameters (Figure 1). Intraclass correlation coefficients for the absolute agreement were 0.93 (95% CI: 0.91-0.95; *P* < 0.001) in calcification angle, 0.89 (95% CI: 0.86-0.92; *P* < 0.001) in calcification thickness, and 0.94 (95% CI: 0.89-0.96; *P* < 0.001) in calcification length. The AI and visual assessments agreed on recommendations to perform lesion modification (defined as calcium score >3^1^) in 215 of 227 lesions (94.7%; Kappa = 0.85; *P* < 0.001). The Kaplan-Meier estimates of 1-year target lesion failure (a composite of cardiac death, target-vessel myocardial infarction, and clinically indicated target lesion revascularization) were 12.8% and 8.4% in AI calcium score >3 and 0 to 3, respectively (log-rank *P* = 0.701).

This is the first study to report the quantitative accuracy of commercially available AI assessment system compared with visual assessment of calcification visualized through OCT in the real-world clinical setting. Overall, AI showed excellent agreement with visual assessment. Moreover, binary calcium score categorization by AI agreed almost perfectly with that performed by visual assessment, suggesting that AI would aid decision-making regarding whether to perform lesion modification before stenting. However, our analysis suggested that the current AI system might have overestimated calcification in acute coronary syndrome lesions where the lipid component is prevalent in general. Although the rate of target lesion failure at 1 year was numerically higher in lesions with AI calcium score >3 compared with AI calcium score 0 to 3, the difference was not significant due to the limited number of patients included in the study. Further prospective multicenter studies are warranted to evaluate the accuracy of AI OCT assessment of calcified plaque and its impact on clinical outcomes.
